# EEG signatures change during unilateral Yogi nasal breathing

**DOI:** 10.1038/s41598-021-04461-8

**Published:** 2022-01-11

**Authors:** Imran Khan Niazi, Muhammad Samran Navid, Jim Bartley, Daniel Shepherd, Mangor Pedersen, Georgina Burns, Denise Taylor, David E. White

**Affiliations:** 1 BioDesign Lab, School of Engineering, Computer and Mathematical Sciences, Auckland University of Technology, Auckland, New Zealand; 2 New Zealand College of Chiropractic, Auckland, New Zealand; 3 Department of Health Science and Technology, Aalborg University, Aalborg, Denmark; 4 School of Psychology & Neuroscience, Auckland University of Technology, Auckland, New Zealand; 5 Rehabilitation Innovation Centre, Health & Rehabilitation Research Institute, AUT University, Auckland, New Zealand

**Keywords:** Electrophysiology, Stress and resilience, Brain injuries, Stroke, Biomedical engineering, Translational research

## Abstract

Airflow through the left-and-right nostrils is said to be entrained by an endogenous nasal cycle paced by both poles of the hypothalamus. Yogic practices suggest, and scientific evidence demonstrates, that right-nostril breathing is involved with relatively higher sympathetic activity (arousal states), while left-nostril breathing is associated with a relatively more parasympathetic activity (stress alleviating state). The objective of this study was to further explore this laterality by controlling nasal airflow and observing patterns of cortical activity through encephalographic (EEG) recordings. Thirty subjects participated in this crossover study. The experimental session consisted of a resting phase (baseline), then a period of unilateral nostril breathing (UNB) using the dominant nasal airway, followed by UNB using the non-dominant nasal airway. A 64-channel EEG was recorded throughout the whole session. The effects of nostril-dominance, and nostril-lateralization were assessed using the power spectral density of the neural activity. The differences in power-spectra and source *localization* were calculated between EEG recorded during UNB and baseline for delta, theta, alpha, beta and gamma bands. Cluster-based permutation tests showed that compared to baseline, EEG spectral power was significantly (1) decreased in all frequency bands for non-dominant nostril UNB, (2) decreased in alpha, beta and gamma bands for dominant nostril UNB, (3) decreased in all bands for left nostril UNB, and (4) decreased in all bands except delta for right nostril UNB. The beta band showed the most widely distributed changes across the scalp. our source localisation results show that breathing with the dominant nostril breathing increases EEG power in the left inferior frontal (alpha band) and left parietal lobule (beta band), whereas non-dominant nostril breathing is related to more diffuse and bilateral effects in posterior areas of the brain.These preliminary findings may stimulate further research in the area, with potential applications to tailored treatment of brain disorders associated with disruption of sympathetic and parasympathetic activity.

## Introduction

Over 50 years ago, Kleitman hypothesized that an ultradian Basic Rest-Activity Cycle (BRAC) entrained some functions of the central nervous system^1^. For example, during sleep, the BRAC manifests itself in the cyclic pattern of non-rapid and rapid eye movement stages. Similarly, the nasal cycle, manifesting rhythmic changes in left and right nasal airflow, is an ultradian oscillation representing a quantifiable shift of lateralised autonomic function. This nasal cycle was first described by Kayser, though the regulative mechanisms, period, and patterns of the cycle require further study^2^. Further questions have been asked about the function of the nasal cycle and whether the different poles of the cycle relate to unique autonomic, cerebral, and functional states^3^. Some authors have extended the BRAC hypothesis to include a tight coupling of the nasal cycle to the ultradian rhythm of alternating cerebral dominance, where right nostril-left hemisphere dominance and left nostril-right hemisphere dominance are linked to the activity and rest phases of the BRAC respectively^4,5^.

The nose consists of two anatomically separate passageways, each with a unique blood supply and nerve endings. Blood circulation to each nasal conchae serves to actuate the nasal cycle^6,7^, and supply thermal energy and fluid for humidification in order to condition inhaled air^8^. The erectile venous tissue of the nasal mucosa enables the nasal conchae to congest and decongest, influencing airflow^3^. During decongestion, or the ‘working phase’, the air is moistened and warmed, and airflow is increased. Concurrently, the contralateral nostril is more congested with comparatively reduced airflow and moisture, occupying the ‘resting phase’ of the nasal cycle^7^. However, beyond its primary respiratory functions, some have proposed that the nasal cycle is coupled to higher-order brain processes as well as the other major bodily systems (neuroendocrine, cardiovascular, fuel-regulatory, immune, and gastrointestinal) that are regulated by the autonomic nervous system via the hypothalamus^4,9^. If this is true, then airflow privileging the left nostril only should be associated with unique biological and psychological states when compared to airflow privileging the right nostril only.

The notion that nasal airflow is associated with unique states has a historical precedent. In ancient India, Yogis proposed that a technique called “unilaterally forced nostril breathing” (UNB) leads to selective unilateral activation of the opposing cerebral hemisphere^3,9^. The UNB technique typically involves plugging one nostril while air is vigorously forced through the other nostril. The tantric text “Shiva Swarodaya” (*Swara yoga*) focused on different modes of breathing and their influence on somatic and psychological processes^10^. It described three types of ‘swara’s’ emerging from the ‘nadis’, where the Nadi represent physiological channels giving passage to various ‘energies’. The three types of Swara’s are:

1. Flow from the left nostril, ‘Chandra anulomaviloma’, which produces a calming and relaxing effect on the body.
2. Flow from the right nostril, ‘Surya anulomaviloma’, which is associated with activation and high-energy states.
3. Flow from both nostrils, representing the transient state between left and right breathing.

In more modern times, Shannahoff-Khalsa’s work on the nasal cycle has supported the postulates of the ancient Yogis, albeit with a more scientific approach^5^. Using both electroencephalograms (EEG) and magnetoencephalography (MEG), it was demonstrated that UNB increases activity in the contralateral hemisphere. Consistent with the MEG and EEG results, further findings using behavioural measures indicated that spatial and verbal cognition was enhanced with left and right nostril breathing respectively^11,12^. Shannahoff-Khalsa proposed that UNB stimulates the contralateral hemisphere and facilitates its resident cognitive functions^5^, with Singh et al. reporting a link between cerebral blood flow and nasal dominance, with right-nostril breathing associated with greater blood flow in the left frontal cortex^13^. Similarly, right-nostril breathing was found to facilitate the activity of the left hemisphere, as measured using event-related potentials^14^.

Foster et al. describe a ‘functional cerebral systems’ model in which cerebral activity is not only balanced along a lateral (left–right) axis but also longitudinally (anterior vs. posterior)^15^. They argued that the anterior and posterior regions of a hemisphere exist in a reciprocally balanced and complementary relationship, such that when one is activated the other is partially suppressed. Likewise, a lateral axis exists, whereby activity on one side suppresses activity on the other. The longitudinal axis has been neglected in UNB research, which hitherto has measured the differences in total EEG power between the hemispheres. Thus, several competing hypotheses emerge when considering the relationship between cerebral activity and nasal breathing. The first hypothesis is the hypothesis of no effect in which nasal breathing has no relationship with cerebral activity. The second hypothesis comes from the three types of Swara’s and Shannahoff-Khalsa’s data, in which a contralateral bias in hemispheric activity would be seen according to the dominant nostril^5^. Following on from the ancient yogis, it could also be hypothesized that both hemispheres would be equally aroused during right nasal breathing, which supposedly induces an excited state, and equally attenuated with left nasal breathing, which induces a calming state. Finally, hypothesis four comes from the data of Singh et al. and the model of Foster et al., the latter whom would argue that if right UNB leads to greater activation of the left prefrontal region then a subsequent decrease in left posterior activity will occur, which in turn will serve to increase activity in the right posterior region and by turn decrease activity in the right frontal region^13,15^. Note that the same logic applies where the right prefrontal region was to experience greater activation.

In summary, the flow of air through the left and right nostrils may have differing effects on higher-order cerebral processes. The current study seeks to test the first three hypotheses mentioned above, and should any be supported, then further research in the applicability of UNB at the clinical level should be pursued.

## Methods

### Participants

Thirty participants, 19 females and 11 males were recruited for the study ranging in age from 20 to 53 years. The participant's details are given in Table 1. The study was approved by the AUT Ethics Committee (AUTEC, approval number 14/02). The study was conducted following the Declaration of Helsinki. The subjects gave their written informed consent to participate in the study. Following criteria were used for the exclusion of the participants: recent upper-airway or sinus infection; a history of mental illness; presence of a respiratory condition or allergy, and current smokers and ex-smokers of less than 5 years.

**Table 1:** Participants characteristics.

	n	Percent (%)
**Sex**		
Female	19	63
Male	11	37
**Age range**		
20–29	13	43
30–39	10	33
40–49	6	20
50–59	1	3
**Smoking status**		
Never	26	87
Ex 5–9 years	2	7
Ex 10–20 years	1	3
Ex 20+ years	1	3
**Dominant handiness**		
Right	29	97
Left	0	0
Ambidextrous	1	3
**Respiratory Health**		
Good	30	100
Screened out of the study	0	0

### Data acquisition

Cortical activity was recorded using a 64-channel EEG using NuAMPs (Compumedics Neuroscan, Inc., Charlotte, NC, USA) at a sampling rate of 500 Hz. The ground electrode was placed next to the Cz. The impedance was kept below 5 kΩ. During the EEG data collection, the participants were asked to pay attention to a silent film and advised that there would be a short test at the end of the collection. A silent film was chosen to remove the participant’s focus from their breathing pattern and style, so the related test results had no implication. This distraction was to remove the effect that the focus, or mindfulness, of their breathing, might have on increasing brain activity, as shown by Moore et al.^16^.

**Figure 1:**
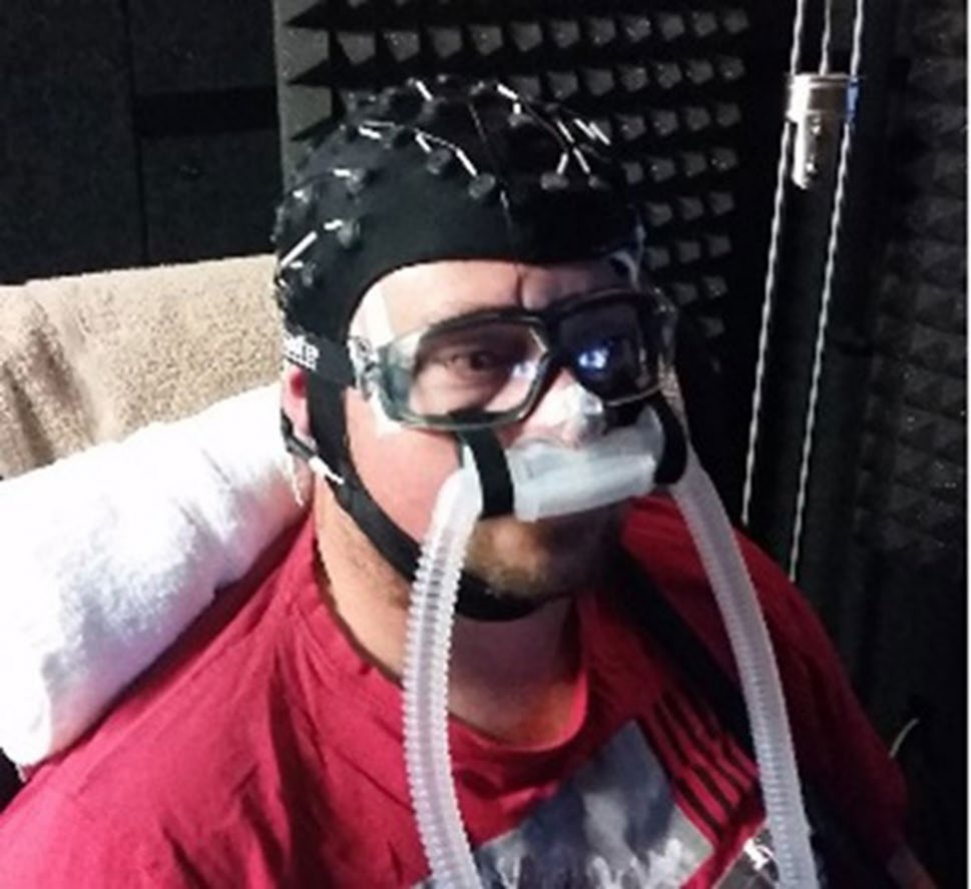
n-CPAP and EEG cap setup.

Each participant breathed pressurised ambient air delivered by a new type of nasal Continuous Positive Airway Pressure (n-CPAP) device, called the Rest-Activity-Cycler (RACer), developed at the BioDesign Lab, AUT University. This device delivers pressurised ambient air to the spontaneously breathing participant, with flow apportioned to bias a greater amount of airflow through one nostril. The nasal mask size was selected for each participant regarding the size of the nostrils and attached to goggles to hold it in place on the participant’s face (Fig. 1). Informed consent for publishing the image of the participants was taken.

Independent control of the air flowing through each nostril during tidal breathing is realised by the nasal mask having a divider between each nostril, enabling two separate pneumatic pathways to deliver different pressures to each side of the nose. The RACer’s software settings (Table 2) was pre-set to enable airflow sensed during the first five breaths while breathing ambient air pressurised to 4 cmH_2_O to determine the user’s endogenous status of nostril dominance as part of the normal nasal cycle, where each side of the nose periodically take turns in conducting the greater airflow.

**Table 2:** RACer software settings used.

RACer setting	Unit	Value
titPres	cmH_2_0	8
reqMeasBreaths	Breaths	5
rampATime	min	3
desInhAv	%	90
desExhAv	%	10
Steady	min	10
Swap	min	5
Steady	min	10
Swap	min	5

**Figure 2:**
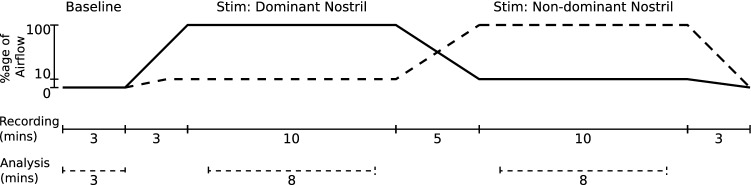
Stimulation and analysis overview.

Once the natural nasal airflow bias had been determined at the start of testing, the air pressure was then gradually increased to a titration pressure of 8 cmH_2_O (titPres) over 3 min (rampATime). During this period the percentage of airflow biased to the dominant nostril (desInhAv) was preset to 90%, the maximum provided by the RACer system, to provide near unilateral nostril breathing (UNB). The non-dominant nostril airflow (desExhAv) was correspondingly pre-set to 10%. Change in the side of the nose passing the greater airflow was regulated by the RACer software which had two pre-set conditions; the period where no change in the side of the nose passing the dominant airflow occurs (Steady) and where a progressive switch in airflow apportionment between each side of the nose occurs (Swap). With these settings, once the RACer system is up to full pressure, it maintains its initial UNB airflow bias for 10 min before progressively swapping dominant sides of the nose over a subsequent 5-min period. This reversal in UNB airflow bias between each side of the nose was then maintained for a further 10 min. Figure 2 presents the spectral plots of temporal combination of participant UNB breathing status and EEG data collection protocols.

### Experimental protocol

Before the data collection, participants attended an introductory information session, and underwent a nasal airways examination. The examination was undertaken using a nasal speculum and head-torch and visual assessment of the participant’s nasal cavity. Exclusion criteria included any noticeable septum deviation or perforation, fresh or dried blood present, relative to normal (dull, moist, smooth, and clean) mucous membranes.

On commencement of data collection, participants had the EEG cap applied while seated in a recliner chair, set so they had their hips at 100°, knees at 0°, and ankles relaxed in slight plantarflexion. They were then instructed to focus on a silent film throughout testing before the nasal mask was applied, and EEG data were collected for the initial 3-min period. During this time, the participant was instructed to breathe normally while the nasal mask on but not pneumatically connected to the RACer system (baseline). Then the RACer device was turned on, and the pre-set program of UNB control started. This program began by identifying the dominant nostril by monitoring the initial five breaths. Then a pressure ramp-up period slowly increased the pressure so that the majority of airflow passed through their naturally dominant nostril, simulating near UNB conditions. Across participants, this served to randomise the order of the sides of the nose initially receiving UNB, and this condition was maintained for 10 min, over which EEG data was collected. From here, the RACer system regulated the swap in airflow bias between each side of the nose over 5 min, reversing the side of the nose receiving UNB. This reversal inside the nose receiving UNB was maintained for a further 10 min over which EEG data was collected.

### EEG processing

#### Preprocessing

The EEG was processed offline using scripts developed in MATLAB 2015b (The MathWorks, Inc., Natick, MA, USA.) and functions from toolboxes EEGLAB version 14.1.1^17^, ERPLAB version 6.1.4^18^, FieldTrip version 20180912^19^. The preprocessing pipeline has been used previously for evoked potentials and resting-state EEG^20,21^. The mastoid channels (M1 and M2) were removed and not used for further analysis. The noisy channels in the raw EEG were identified using the PREP pipeline version 0.55.1^22^. Additionally, the PREP pipeline was used for removing mains noise and obtaining the average reference. For artefact rejection, the continuous PREPed data was zero-phase filtered with a 2nd order high-pass Butterworth filter with a cut-off frequency of 1 Hz. Afterward, the data was divided into 0.5 s long epochs. An epoch with artefact was identified if one or more of the following parameters were true for any channel: (1) absolute voltage greater than 100 μV, (2) peak-to-peak voltage greater than 150 μV in a 200 ms wide sliding window with a step size of 100 ms, (3) sample-to-sample difference of more than 50 μV, and (4) absolute voltage less than 1 μV for 150 ms. Afterwards, a manual inspection of all the epochs was done. The epochs with step-like artefacts (identified by the voltage above 100 μV given by a step-function with a 200 ms long sliding window having a step size of 50 ms) in the frontal channels were not removed as the artefact corresponded to the eye-blinks and eye-movements^23^.

After identification of noisy epochs, independent component analysis (ICA) was used to remove non-brain components (such as eye blinks, muscle activity and noise from channels and mains) from the data. The 1 Hz high-pass filtered PREPed EEG (noisy channels removed) was downsampled to 250 Hz to reduce ICA computational time, segmented into 0.5 s long epochs and bad epochs identified above were removed. The adaptive mixture ICA (AMICA) algorithm was used since it has been shown that its performance is superior to that of other ICA algorithms^24^. The ICs were marked as brain or non-brain components based on their spatial distributions (topographies), time courses, spectrograms, event-related potential images and equivalent current dipole models. The cleaned EEG was obtained by applying the ICA matrix to the PREPed dataset which was zero-phase 0.5–200 Hz 2nd order Butterworth filtered with bad epochs removed. The noisy channels were interpolated using the spherical method implemented in EEGLAB. The cleaning procedure was performed on the 3 min of baseline block and the middle 8 min of the steady-state blocks.

Next, the EEG data was transformed and saved in two ways for further analysis:

1. The data of participants with dominant left nostril was mirrored in the sagittal plane. This way it was assumed that every subject had a dominant right nostril. This was done to answer the question: are breathing effects based on dominance.
2. The segments were ordered to analyze data for the right and left nostril stimulation irrespective of the nostril dominance. This data was used to answer the question: does lateralized nasal breathing affect EEG.

#### Spectral analysis

The spectral analysis was performed on data (segmented into 4 s long epochs) from each block (baseline, dominant nostril, non-dominant nostril) which had at least 80% clean EEG (i.e. 2.4 min of clean EEG from the baseline blocks, and 6.4 min of clean EEG each from the stimulation blocks). For any block having more clean data than the minimum required, 36 epochs for the baseline and 96 epochs for the stimulation blocks were randomly selected. The power spectra were calculated between 1 and 80 Hz using the Fourier basis with a 4 s long Hanning window. Afterward, the average power of each classical frequency band (delta (1–4 Hz), theta (4.1–8 Hz), alpha (8.1–12 Hz), beta (12.1–32 Hz), and gamma (32.1–80 Hz)) was computed.

#### Source localization

The localization of activity in the brain was estimated in the LORETA-KEY software, version 20151222^25^ (freely available at www.uzh.ch/keyinst/loreta). For source localization, we used sLORETA, which is a linear inverse algorithm that estimates the distribution of cortical generators of the EEG in three-dimensions, with lowest localization error compared to several other linear inverse methods^25^. The sLORETA implementation uses a reference brain from the Montreal Neurological Institute (average MRI brain map (MNI-152))^26^ with cortical gray matter divided into 6239 voxels with a resolution of 5 mm. For sLORETA analysis, the EEG was segmented into 8 s long epochs to obtain smooth power spectral density. Similar to the criteria for spectral analysis, data from subjects with at least 2.4 min of clean EEG from the baseline blocks, and 6.4 min of clean EEG each from the stimulation blocks were used. For any block with more than 80% clean data epochs were randomly selected to reduce the data length to 2.4 min (baseline blocks) or 6.4 min (stimulation blocks). The sLORETA was performed in the frequency domain where cross-spectral matrices for each subject were computed in the LORETA-KEY software for the same five frequency bands as in the power spectral analysis above. Subsequently, the cross-spectral matrices were averaged for each subject and used as input for the sLORETA.

### Statistical analysis

Non-parametric cluster-based permutation tests^27^ were used to investigate the difference in the power spectrum between

1. For effects of dominance (mirrored data)
  (i) non-dominant stimulation and baseline blocks
  (ii) dominant stimulation and baseline blocks
2. For effects of lateralization (non-mirrored data)
  (i) left stimulation and baseline blocks
  (ii) right stimulation and baseline blocks.

The clusters were defined as two or more channel-frequency pairs each with *p* < 0.05 from the paired two-tailed *t*-test. The test statistic was defined as the maximum of cluster-level statistics obtained by adding the *t*-values within each cluster. A cluster was considered significant if its Monte Carlo probability for each tail exceeded the threshold of 0.00625 (Bonferroni corrected: (0.05/4)/2) compared to the reference distribution which was approximated by the Monte Carlo method with 5000 permutations.

The statistical procedure for source localization of EEG was performed in the LORETA-KEY software using non-parametric mapping^28^, which utilized Fisher’s random permutation test with 5000 randomizations to control for multiple comparison problem. The paired two-tailed t-test was used to find differences in the current sources across the different frequency bands. Similar to the spectral analysis, the tests were used to identify differences in brain activity between non/dominant stimulation and baseline blocks, and left/right stimulation and baseline blocks.

## Results

### Effects of nostril dominance

**Figure 3:**
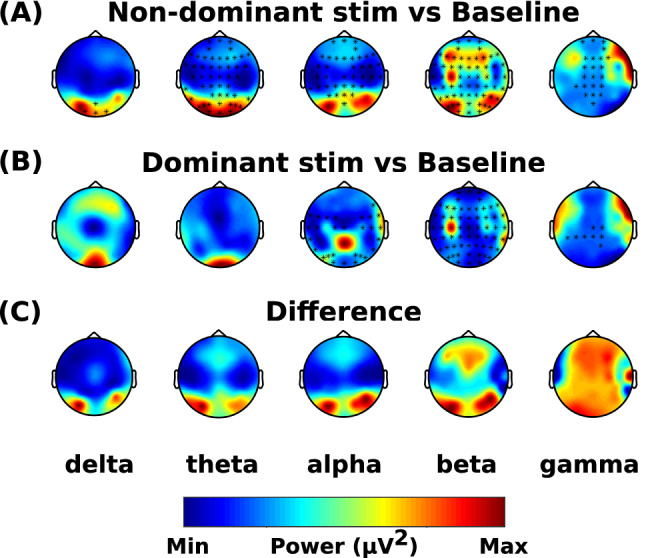
Effects of dominance. The data was mirrored in the sagittal plane for left stimulations, so it was assumed that every subject received stimulation to their right dominant nostril. The difference of spectral power is shown for (**A**) non-dominant nostril stimulation vs baseline (non-dominant nostril stimulation minus baseline), (**B**) dominant nostril stimulation vs baseline (dominant nostril stimulation minus baseline), and (**C**) shows the difference between (**A**) and (**B**) ((**A**) minus (**B**)). Asterisks represent significant clusters.

The spectral power in response to non-dominant nostril stimulation was significantly different in all bands compared to baseline (1 positive cluster, *p* < 0.001) (Fig. 3A). The spectral power in response to dominant side stimulation was significantly different in alpha, beta and gamma bands compared to the baseline block (1 positive cluster, *p* < 0.001) (Fig. 3B)). Compared to dominant side stimulation, the non-dominant side stimulation increased the activity in the gamma band, however, there was a decrease in the spectral power in all other bands across the scalp except in the posterior region where it was similar or higher (Fig. 3C).

**Figure 4:**
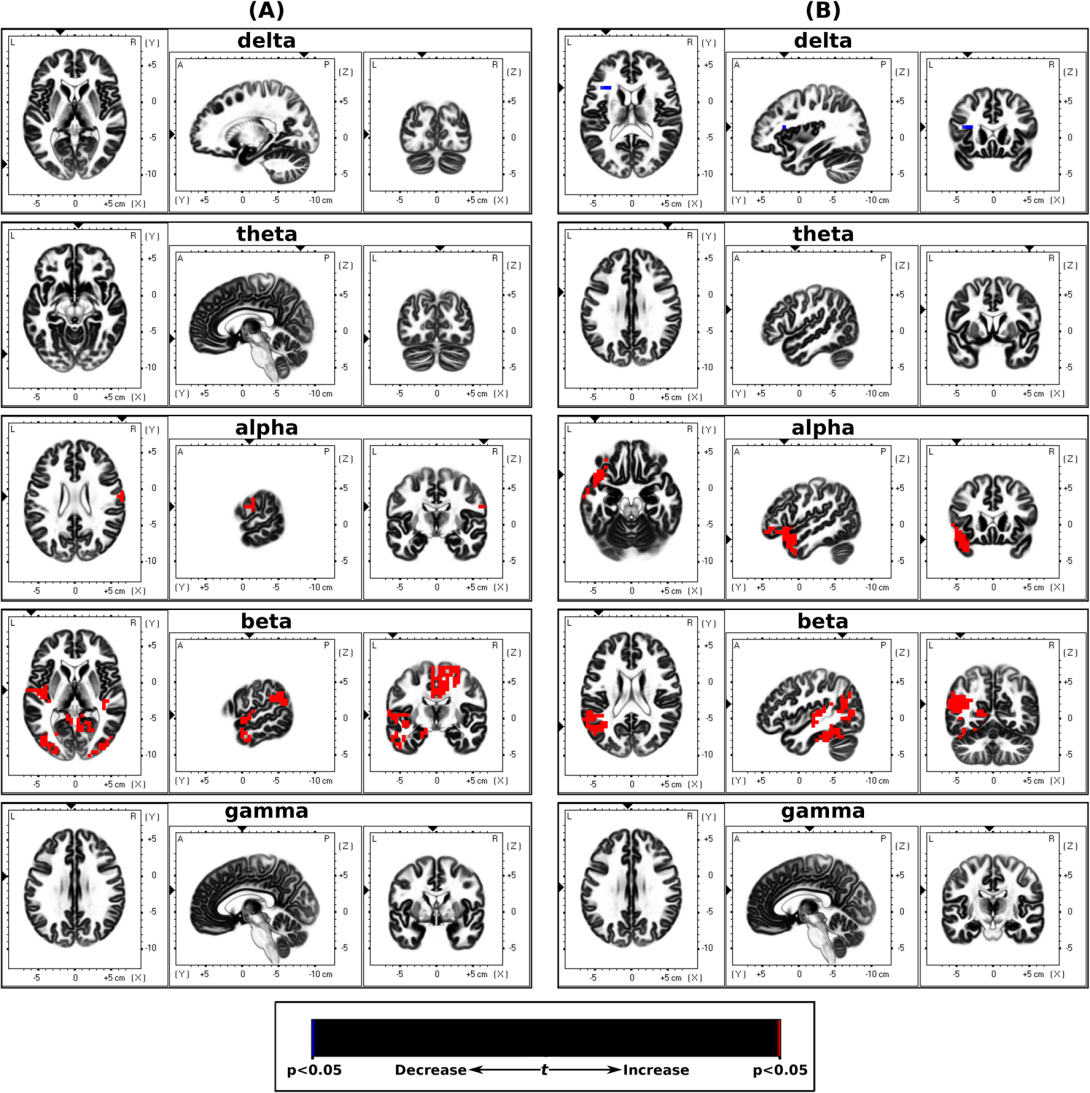
Dominance sLORETA. Slice views of source locations with the changes in activity (t-values) for each frequency between (**A**) non-dominant stimulation and baseline EEG for each frequency band, and (**B**) dominant stimulation and baseline EEG. Significant differences (p < 0.05) in activity are shown in blue and red colors.

The sLORETA analysis between EEG during non-dominant nostril stimulation and baseline blocks showed a significant differences in cortical activity in the delta, alpha and beta bands, whereas differences in the alpha and beta bands were seen between the dominant nostril stimulation and baseline blocks. The results from the sLORETA analysis are summarized in Tables 3 and 4 and Fig. 4.

**Table 3:** sLORETA localized EEG cortical sources with significant differences between the non-dominant stimulation and baseline states.

Brain structure (broadmann area)	Delta (1–4 Hz)	Theta (4–8 Hz)	Alpha (8–12 Hz)	Beta (12–32 Hz)	Gamma (32–80 Hz)
Angular gyrus (39)				9	
Anterior cingulate (24, 25, 32, 33)				36	
Cingulate gyrus (6, 23, 24, 31, 32)				146	
Cuneus (18, 30)				8	
Fusiform gyrus (19, 20, 36, 37)				114	
Inferior frontal gyrus (10, 45, 46, 47)			9		
Inferior occipital gyrus (18, 19)				11	
Inferior parietal lobule (39, 40)				10	
Inferior temporal gyrus (19, 20, 23, 37)				21	
Insula (13)				23	
Lingual gyrus (18, 19)				61	
Medial frontal gyrus (6, 8, 9, 32)				118	
Middle frontal gyrus (6, 8, 46, 47)			2	43	
Middle occipital gyrus (18, 19, 37)				57	
Middle temporal gyrus (19, 21, 22, 37, 39)				54	
Paracentral lobule (4, 5, 6, 31)	s			49	
Parahippocampal gyrus (19, 27, 28, 30, 34, 35, 36, 37)				140	
Postcentral gyrus (2, 3, 4, 5)			6	12	
Posterior cingulate (23, 29, 30, 31)				27	
Precentral gyrus (4, 6)			3	23	
Precuneus (7)				1	
Sub-gyral (6, 8, 19, 20, 21)				15	
Superior frontal gyrus (6, 8)				119	
Superior occipital gyrus (19)				1	
Superior temporal gyrus (13, 21, 22, 38, 39, 41)				42	
Supramarginal gyrus (39, 40)				18	
Transverse temporal gyrus (41, 42)				8	

The number of voxels with significant power changes (p < 0.05) is listed.

**Table 4:** sLORETA localized EEG cortical sources with significant differences between the dominant stimulation and baseline states.

Brain structure (broadmann area)	Delta (1–4 Hz)	Theta (4–8 Hz)	Alpha (8–12 Hz)	Beta (12–32 Hz)	Gamma (32–80 Hz)
Angular gyrus (39)				9	
Cuneus (30)				1	
Fusiform gyrus (19, 20, 36, 37)			1	69	
Inferior frontal gyrus (45, 47)			28		
Inferior parietal lobule (40)				4	
Inferior temporal gyrus (20, 21, 37)			2	15	
Insula (13)	2			7	
Lingual gyrus (18, 19)				10	
Middle frontal gyrus (10, 11, 47)			15		
Middle occipital gyrus (19, 37)			7		
Middle temporal gyrus (21, 22, 37, 39)			22	41	
Parahippocampal gyrus (19, 27, 28, 30, 35, 36, 37)				61	
Posterior cingulate (29, 30, 31)				19	
Precuneus (39)				1	
Sub-gyral (19, 37, 39)				4	
Superior temporal gyrus (13, 21, 22, 38, 39, 31,42)			36	67	
Supramarginal gyrus (39, 40)				15	
Transverse temporal gyrus (41, 42)				9	

The number of voxels with significant power changes (p < 0.05) is listed.

### Effects of lateralization

**Figure 5:**
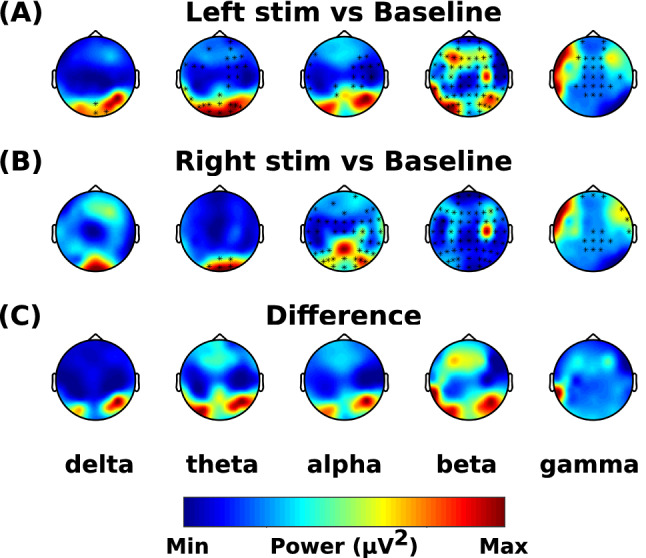
Effects of lateralization. The data was analyzed irrespective of the dominant nostril. The right and left side stimulations across subjects were combined irrespective of the order they were provided. The difference of spectral power is shown for (**A**) left nostril stimulation vs baseline (left nostril stimulation minus baseline), (**B**) right nostril stimulation vs baseline (right nostril stimulation minus baseline), and (**C**) shows the difference between (**A**) and (**B**) ((**A**) minus (**B**)). Asterisks represent significant clusters.

The spectral power in response to left side stimulation was significantly different in all bands compared to the baseline (1 positive cluster, *p* < 0.001) (Fig. 5A). The spectral power in response to right side stimulation was significantly different in theta, alpha, beta and gamma bands compared to the baseline block (1 positive cluster, *p* < 0.001) (Fig. 5B). Compared to the right side stimulation, the left side stimulation decreased the spectral power in delta, theta and alpha bands across the scalp except in the posterior region where it was similar or higher. In the gamma band, the power across the whole scalp was lower when left side stimulation was compared with right side stimulation (Fig. 5C).

**Figure 6:**
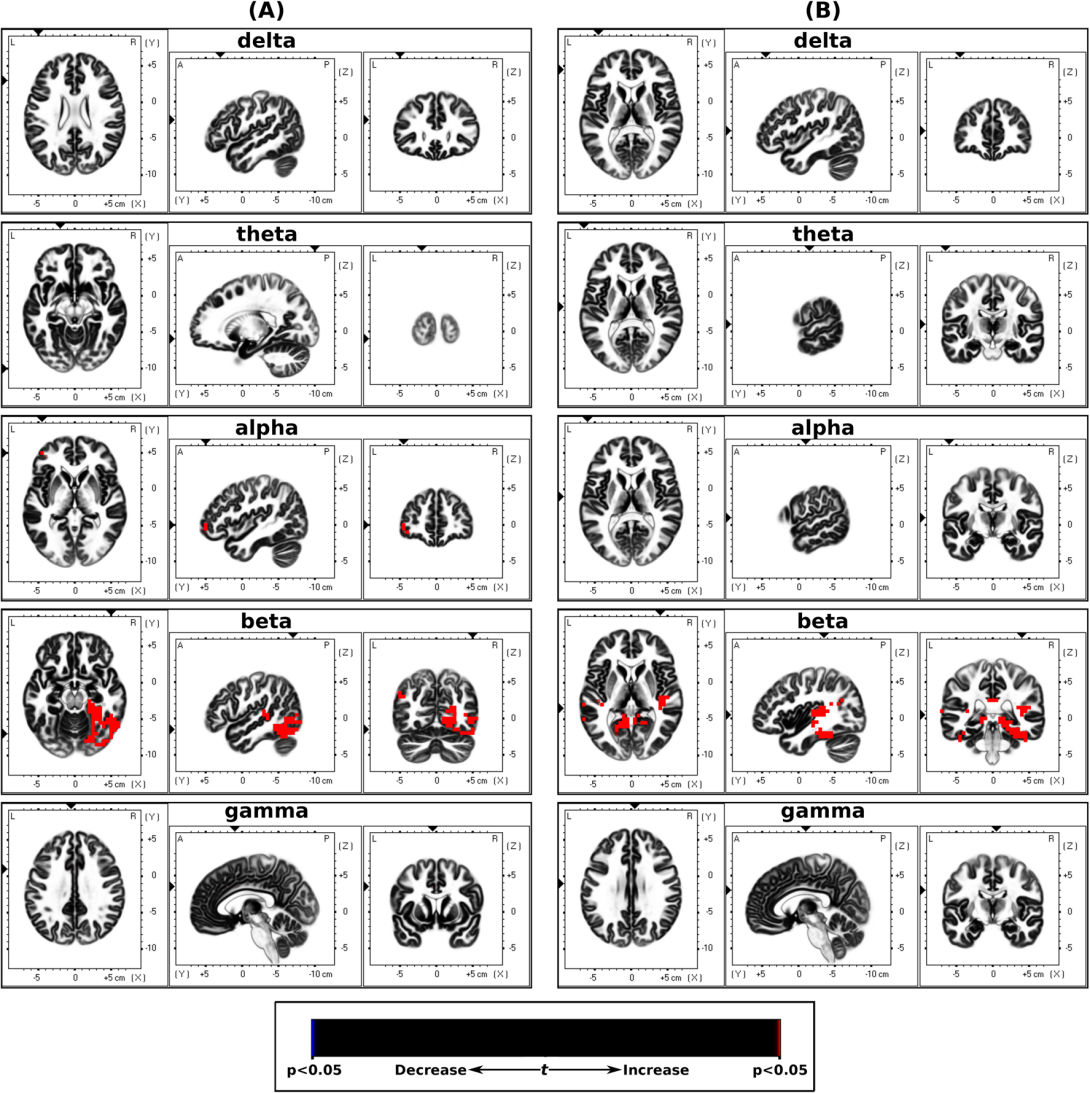
Laterization sLORETA. Slice views of source locations with the changes in activity (t-values) for each frequency band between (**A**) left nostril stimulation and baseline EEG, and (**B**) right nostril stimulation and baseline EEG. Significant differences (p < 0.05) in activity are shown in blue and red colors.

The sLORETA analysis between EEG during left stimulation and baseline blocks showed a significant increase in cortical activity in the alpha and beta bands, whereas the activity increased in the beta band for the right stimulation vs baseline block. The results from the sLORETA analysis are summarized in Tables 5 and 6 and Fig. 6.

**Table 5:** sLORETA localized EEG cortical sources (laterlised) with significant differences between the left nostril stimulation and baseline states.

Brain structure (broadmann area)	Delta (1–4 Hz)	Theta (4–8 Hz)	Alpha (8–12 Hz)	Beta (12–32 Hz)	Gamma (32–80 Hz)
Angular gyrus (39)				8	
Anterior cingulate (24, 32)				31	
Cingulate gyrus (6, 32)				5	
Cuneus (17, 18, 30)				9	
Fusiform gyrus (18, 19, 20, 37)				69	
Inferior frontal gyrus (10)			1		
Inferior occipital gyrus (18, 19)				14	
Inferior temporal gyrus (19, 20, 37)				17	
Insula (13)				2	
Lingual gyrus (17, 18, 19)				47	
Medial frontal gyrus (6, 8, 9)				34	
Middle frontal gyrus (6, 8, 10, 11)			4	9	
Middle occipital gyrus (18, 19, 37)				45	
Middle temporal gyrus (19, 21, 22, 37, 39)				39	
Paracentral lobule (4, 5, 6)				28	
Parahippocampal gyrus (19, 27, 28, 30, 35, 36, 37)				60	
Postcentral gyrus (3, 4, 5, 7)				24	
Posterior cingulate (29, 30, 31)				30	
Precentral gyrus (4, 6)				14	
Precuneus (31)				1	
Sub-gyral (6, 8, 37)				4	
Superior frontal gyrus (6, 8)				22	
Superior occipital gyrus (19)				1	
Superior temporal gyrus (13, 22, 39, 41, 42)				19	
Supramarginal gyrus (39, 40)				2	
Transverse temporal gyrus (41)				3	

The number of voxels with significant power changes (p < 0.05) is listed.

**Table 6:** sLORETA localized EEG cortical sources (laterlised) with significant differences between the right nostril stimulation and baseline states.

Brain structure (broadmann area)	Delta (1–4 Hz)	Theta (4–8 Hz)	Alpha (8–12 Hz)	Beta (12–32 Hz)	Gamma (32–80 Hz)
Angular gyrus (39)				15	
Cingulate gyrus (23, 31)				4	
Cuneus (18, 30)				2	
Fusiform gyrus (19, 20, 36, 37)				65	
Inferior parietal lobule (40)				1	
Inferior TEMPORAL GYRUS (20, 37)				4	
Insula (13)				13	
Lingual gyrus (18, 19)				24	
Middle occipital gyrus (19)				1	
Middle temporal gyrus (19, 21, 22, 37, 39)				28	
Parahippocampal gyrus (19, 20, 27, 28, 30, 35, 36, 37)				102	
Posterior cingulate (18, 23, 29, 30, 31)				44	
Precuneus (39)				1	
Sub-gyral (19)				1	
Superior temporal gyrus (13, 21, 22, 39, 41, 42)				47	
Supramarginal gyrus (39, 40)				18	
Transverse temporal gyrus (41)				7	

The number of voxels with significant power changes (p < 0.05) is listed.

## Discussion

In this study, we assessed the EEG activity associated with applied unilaterally forced nostril breathing (UNB), from the perspectives of the left–right lateral axis and the anterior–posterior longitudinal axis. The results showed that the non-dominant airway UNB decreased the neural activity in all bands, however, dominant airway UNB only decreased activity in higher frequency bands. But overall, in the delta, theta, alpha, and beta bands, the dominant airway UNB elicited higher activity compared to non-dominant airway UNB in the frontal and central regions, and lower activity in posterior regions. However, in the gamma band, non-dominant airway UNB elicited higher activity compared to dominant airway UNB. This was also supported by our source localisation results aiming to model specific brain regions associated with UNB (see Fig. 4 and 6). Our source localisation results showed that dominant airway UNB is related to brain changes in areas confined to the left hemisphere (frontal and parietal), whereas non-dominant breathing had more diffuse bilateral effects across posterior and central cortices.

When lateralization was considered, the left airway UNB decreased the brain activity, however, the right airway UNB decreased the activity in all bands except the delta band. Overall, right side airway UNB elicited higher activity compared to left airway UNB in all frequency bands across the whole scalp except in posterior areas. As such, our results do not fully correspond to a previous study that investigated EEG changes during forced alternate nostril breathing^29^. Concordant with our findings, Stanacak and Kuna did find increased alpha/beta power during forced alternate nostril breathing, but they found no EEG differences between left and right nostril expiration (contrary with our study)^29^. In this context, our study adds to the limited research into elucidating the effects of nasal breathing laterality and brain function, as our results suggests that general breathing patterns, and specific nostrils, does influence brain activity.

Our main observation was that left-nostril and non-dominant breathing (usually the left-nostril) was associated with greater EEG power in posterior areas of the brain. Increased posterior EEG power has a longstanding history of occurring during eyes-closed conditions, relaxation and restoration^30^. We interpret increased EEG power associated with left-nostril/non-dominant breathing as being indicative that a subject is entering a more restorative and meditative state. For example, Lagopoulos et al. found evidence of increased alpha EEG activity across posterior regions during nondirective meditation^31^, and Huang and Lo previously found that Zen meditation practitioners displayed increased frontal EEG alpha power and posterior EEG beta power, compared to non-meditators^32^. Together with our results, this suggests that breathing patterns may affect cortical functioning.

The posterior cortex is known to activate during resting-states by increasing functional connectivity within the brain’s default mode network^33^. The default mode network consists of the precuneus, posterior cingulate cortex and lateral parietal lobe^34^ and is known to be involved in introspective brain functions and autobiographical memory, and is likely to be an index of a person’s ‘inner world’^35^. And our source localisation results suggests that brain activity increases in lateral parietal cortices during dominant breathing. The functioning of the default mode network is usually quantified with functional MRI, but common breathing artefacts within the low frequencies of functional MRI (breathing rate and functional MRI has similar frequencies) have precluded this type of analysis. We believe that future studies using multi-echo functional MRI can further delineate the role of the default mode network during lateralised nasal breathing. Multi-echo functional MRI essentially acquires three distinct blood-oxygen-level-dependent signals^36^. One of these signals are associated with breathing artefacts and can be omitted from the final analysis, hence it provides an opportunity to quantify spatial brain effects associated with breathing^37^. It is also worth emphasizing that the frequencies of EEG are higher than the frequencies of breathing patterns, which means that our findings represent neurobiological effects of breathing, rather than an artefact from the mechanisms of breathing. Normal paced breathing occurs between 0.16 and 0.33 Hz representing 10–20 breaths per minute^38^, whereas EEG was filtered above this frequency (at 1 Hz).

Turning to the study’s directional hypotheses, our data did not support the hypothesis of no effect in that we found a relationship between nasal breathing and cerebral activity. However, the proposition that a contralateral bias in hemispheric activity with reference to the dominant nostril should be seen was not supported. Instead, support was marshalled for the hypothesis that both hemispheres would be equally aroused during right nasal breathing, which supposedly induces an excited state, and equally attenuated with left nasal breathing, which induces a calming state. This is the salient finding of the study, with left nasal breathing being associated with a prominent posterior rhythm (in all bands apart from gamma), which is associated with a more relaxed state and introspective thinking. Finally, Foster et al.’s^15^ notion that if right UNB leads to greater activation of the left prefrontal region then a corresponding decrease in left posterior activity should occur (and vice versa), was not supported, with the caveat that the current study design was not well suited to directly address this model and thus more research is needed.

## Conclusions

The flow of air through the left and right nostrils appears to have differing effects on cerebral processes. Should the findings of this study be further supported, then a more scientific and clinical approach of brain disorders involving impaired cerebral asymmetries may be possible. While one study has attempted to utilise right UNB to stimulate the left hemispheres of children with autism^39,40^, the affirmation of our findings would indicate that left UNB would in fact be the correct therapeutic approach, assuming the goal was to induce greater states of calm. However, it remains to be determined conclusively if UNB does in fact have a differential anterior versus posterior hemispheric activation affect, or whether the activation across the hemisphere is relatively equal. Additional brain imaging studies can help us better understand these activation patterns, and if different UNB techniques, aside from the simple equal inhalation versus exhalation patterns can alter the anterior and posterior regions of the hemispheres. If focused breathing changes cortical activity, we believe our approach may be trialled as a potential treatment of brain disorders associated with disruption of sympathetic and/or parasympathetic activity.
